# Crossed Wavelet Convolution Network for Few-Shot Defect Detection of Industrial Chips

**DOI:** 10.3390/s25144377

**Published:** 2025-07-13

**Authors:** Zonghai Sun, Yiyu Lin, Yan Li, Zihan Lin

**Affiliations:** 1 School of Automation Science and Engineering, South China University of Technology, Guangzhou 510641, China; a1027345695@163.com (Y.L.); liyan@scut.edu.cn (Y.L.); 2 School of Automation, Nanjing University of Science and Technology, Nanjing 210094, China; lzh123110011112@njust.edu.cn

**Keywords:** defect detection, few-shot learning, wavelet transform convolution, prototype learning

## Abstract

In resistive polymer humidity sensors, the quality of the resistor chips directly affects the performance. Detecting chip defects remains challenging due to the scarcity of defective samples, which limits traditional supervised-learning methods requiring abundant labeled data. While few-shot learning (FSL) shows promise for industrial defect detection, existing approaches struggle with mixed-scene conditions (e.g., daytime and night-version scenes). In this work, we propose a crossed wavelet convolution network (CWCN), including a dual-pipeline crossed wavelet convolution training framework (DPCWC) and a loss value calculation module named ProSL. Our method innovatively applies wavelet transform convolution and prototype learning to industrial defect detection, which effectively fuses feature information from multiple scenarios and improves the detection performance. Experiments across various few-shot tasks on chip datasets illustrate the better detection quality of CWCN, with an improvement in mAP ranging from 2.76% to 16.43% over other FSL methods. In addition, experiments on an open-source dataset NEU-DET further validate our proposed method.

## 1. Introduction

**Figure 1:**
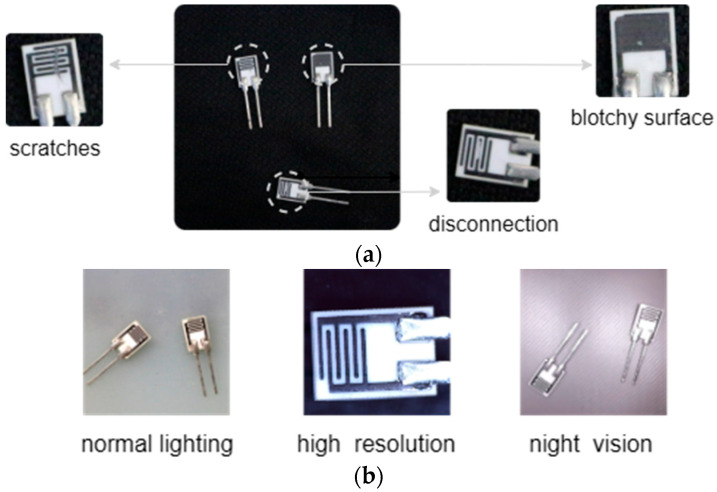
Industrial chip samples. (**a**) Types of defects and (**b**) multiple scenarios of samples.

As the critical component of resistive polymer humidity sensors, the humidity-sensitive resistor chips sense environmental humidity through the change in resistance value. Defects as shown in Figure 1a can directly affect the reliability, where “disconnection” and “blotchy surface” cause open and short circuits, respectively, and “scratches” disable the polymer material on the surface. In this case, defect detection has always been a key research topic in sensor manufacturing. Recently, Automatic Visual Inspection (AVI) based on computer vision and object detection techniques has replaced traditional manual visual inspection. Representative algorithms, such as RCNN-based method [1], SSD variant [2], and YOLO series [3], as well as ViT [4] and SwinT [5] based on the transformer architecture [6], have significantly boosted detection accuracy and efficiency.

Unfortunately, industrial defect samples are usually scarce and scattered. In addition to the sensor manufacturing mentioned in this paper, this situation is common in other precision electronics assembly and high-end component production. Although every production stage is under strict monitoring for high-quality standards, defects caused by unforeseen circumstances would be rare but deadly. Traditional supervised-learning methods relying on abundant labeled samples are ineffective. As a result, few-shot learning (FSL) methods have gained widespread attention and research interest.

Recent years have witnessed impressive progress in FSL for visual tasks and image processing problems, including meta-learning methods (e.g., Meta-AdaM [7], Metadiff [8]), pretraining-finetuning frameworks like Ppt [9], and cross-domain approaches such as CGDM [10]. To improve performance on downstream tasks such as image classification, object detection, and segmentation, researchers attempt to extract highly transferable feature representations from limited annotated samples.

Nonetheless, existing FSL applications in industrial detection still underperform in multi-scene conditions. As illustrated in Figure 1b, defect samples of chips exhibit variations across different conditions. The feature extraction modules struggle to maintain feature consistency for the same defect category in these diverse scenarios, leading to suboptimal detection quality. Our experimental results reveal that, compared to a single-scene dataset, there is a substantial 20–30% performance gap in Mean Average Precision (mAP) when evaluating on a mixed-scene dataset. The detailed analysis will be presented in Section 4.4.1.

To address these limitations, we propose a novel network that introduces two key innovations. First, we develop a dual-pipeline crossed wavelet convolution training mechanism (DPCWC). This module enables parallel feature extraction and cross-pipeline information fusion, effectively capturing discriminative features across multiple scenarios. Second, we design a loss computation module, ProSL, that incorporates feature information from prototypes. This module minimizes the feature distance between identical defect categories across different scenarios, significantly improving the model’s scenario adaptation capability.

The main contributions of our work are summarized below:

- To address performance degradation in industrial chip defect detection under mixed-scene condition (relative to single-scene data), we propose a dual-pipeline feature extractor.
- Our work uses wavelet transform convolution in industrial defect detection. We also adopt a selective strategy to ensure effective but not excessive fusion.
- Unlike existing methods of processing dual-pipeline features, our work employs prototype information to calculate loss value, effectively aligning the features from two pipelines.

## 2. Related Work

### 2.1. Cross-Domain Few-Shot Object Detection

By transferring knowledge from source domain datasets, which contain abundant labeled samples, cross-domain few-shot learning (CD-FSL) aims to address the challenge of data scarcity in target domain.

Compared to image classification [11,12,13,14,15], research on CD-FSL focusing on object detection is relatively limited, but there have been notable works in related fields recently. The work of [16] presents FSCE to learn contrastive-aware object proposal encodings, introducing Contrastive Proposal Encoding (CPE) to improve the performance of few-shot detection. Another approach, Detic [17], trains on detection data and image-labeled data, decoupling classification and localization tasks while developing efficient classification loss functions. In addition, a ViT-based variant [18] demonstrates that a plain Vision Transformer (ViT) without hierarchical structures can achieve competitive detection performance, challenging the conventional reliance on hierarchical backbones.

Particularly in industrial defect detection, the following methods have been of great assistance. (1) The two-stage Fine-tuning Approach (TFA) [19] establishes a classic “pretraining + fine-tuning” paradigm, providing a framework for many CD-FSL applications in industrial detection; (2) DeFRCN [20] addresses the inherent conflict between multi-stage (RPN and RCNN in Faster R-CNN) and multi-task (classification and localization); (3) DE-ViT [21] based on a region-propagation mechanism for localization employs fixed-ratio expansion of proposals, propagated region masks, and the construction of a feature subspace to improve the model’s robustness; (4) CD-ViTO [22] quantifies domain differences across three dimensions: style discrepancy, inter-class variance, and indefinable boundaries. The method achieves superior performance on various datasets.

However, these methods lack specific designs to align defect samples from different scenarios. Insufficient feature interaction manifests poor detection quality in mixed-scene data.

### 2.2. Wavelet Transform

Compared to Fourier Transform, which sacrifices time (or spatial) information to obtain frequency domain information, wavelet transform [23] achieves a good balance between the time (or spatial) and frequency domains. It decomposes signals into wavelet components that simultaneously contain information from both domains above. Therefore, as stated in [24], the unique advantages of the wavelet transform have made it an important tool in the field of signal processing, and it has been widely used in areas such as image processing and industrial supervision.

Research on wavelet transforms and wavelet convolutions has established a solid theoretical foundation and provided practical references for subsequent studies. Notably, Wavelet Compressed Convolution (WCC) [25] demonstrates the viability of the framework “wavelet transforms + neural networks”. Ref. [26] introduces a dual-episode sampling mechanism and breaks new ground to apply wavelet in CD-FSL. In industrial defect detection, ref. [27] presents a dynamic weights-based wavelet attention neural network (DWWA-Net) and achieves remarkable performance improvement.

However, these works addressed a critical challenge in neural network development: the computational inefficiency caused by over-parameterization in traditional CNNs and ViTs when obtaining the global receptive field. In one study [28], the Wavelet Transform Convolution layer (WTConv) effectively resolved this issue while maintaining model performance, representing a significant step forward in efficient feature extraction architectures.

### 2.3. Prototype Learning

The concept of prototype learning was originally introduced in Prototypical Networks [29] and applied to FSL. It establishes class prototypes by computing the mean feature of all samples within each category, and then calculates the distance metric between the query samples and these prototypes for classification.

Subsequent research has significantly enhanced prototype learning through various innovations. For instance, refs. [21,22] extract features through deep convolutional models and develop advanced prototype representations by statistical computations. In [30], the CPLAE model based on self-supervised learning uses category prototypes as anchors for contrastive training. These prototype variants and enhancements have collectively addressed key challenges in prototype learning while expanding its applicability across diverse computer vision tasks.

## 3. Method

### 3.1. Problem Setting

In cross-domain few-shot object detection (CD-FSOD), the source domain dataset $D_{\mathrm{source}}$ and target domain dataset $D_{\mathrm{target}}$ are defined, where $C_{\mathrm{source}}\cap C_{\mathrm{target}}=\emptyset$, i.e., the categories of the two are different. $D_{\mathrm{source}}$ contains numerous labeled data with diverse categories, while $D_{\mathrm{target}}$ has fewer categories and only a small amount of labeled data for each category. The label information includes, but is not limited to, category labels and the coordinates of bounding boxes.

Currently, there are two mainstream approaches to solve the CD-FSOD problem, one is “meta-learning”, and the other is “pretraining + fine-tuning”. Our model adopts the latter, and the main processes are as follows: Firstly, a base model ${Model}_{A}$ is trained in a supervised-learning way using large-scale $D_{\mathrm{source}}$, and subsequently fine-tunes ${Model}_{A}$ using $D_{\mathrm{target}}$ to obtain the specialized ${Model}_{B}$. The fine-tuning process employs an n-way-k-shot sampling strategy from $D_{\mathrm{target}}$, as outlined below:

- The support set $D_{\mathrm{support}}$ for tuning contains k annotated samples for each of n categories.
- The query set $D_{\mathrm{query}}$ for testing comprises unlabeled samples from the same n categories.
- Critical constraints are $D_{s\mathrm{upport}}\cap D_{\mathrm{query}}=\emptyset$ and $C_{s\mathrm{upport}}=C_{\mathrm{query}}$.

This methodology enables effective adaptation of ${Model}_{A}$’s parameters to novel categories on the target domain while maintaining its fundamental detection capabilities. The limited annotated samples (k per category) in $D_{\mathrm{support}}$ provide sufficient supervisory signals for this specialized adaptation, while the disjoint query set ensures a proper evaluation of the model’s generalization performance on unseen target domain instances.

Our research addresses defect detection of a specific type of chip, presenting unique challenges beyond conventional “cross-domain” difficulties. The problem’s complexity manifests through two distinct dimensions: one is significant distributional discrepancies between $D_{\mathrm{source}}$ and $D_{\mathrm{target}}$, and the other is intrinsic variations within the target domain itself arising from illumination changes, and imaging device diversity. Such multi-faceted scenario variations impose stringent demands on model generalization capabilities.

### 3.2. Preliminaries: Wavelet Transform Convolution in Image Field

For an input image X, four specific filters (Equation (1)) based on the 2D Haar wavelet transform [31] are employed to conduct image decomposition and reconstruction. The forward wavelet transform (Equation (2)) decomposes the image into multiple components, while the inverse wavelet transform (Equation (3)) enables image reconstruction. The decomposition yields $X_{\mathrm{LL}}$ representing the low-frequency approximation component that preserves the image’s primary structural information, along with three high-frequency detail components, $X_{\mathrm{LH}}$ (horizontal), $X_{\mathrm{HL}}$ (vertical), and $X_{\mathrm{HH}}$ (diagonal), which collectively capture the image’s edge and texture characteristics.

(1) $$\begin{array}{cccccccc} f_{LL} & =\frac{1}{2}\left[\begin{array}{cc} 1 & 1\\ 1 & 1 \end{array}\right], & f_{LH} & =\frac{1}{2}\left[\begin{array}{cc} 1 & -1\\ 1 & -1 \end{array}\right], & f_{HL} & =\frac{1}{2}\left[\begin{array}{cc} 1 & 1\\ -1 & -1 \end{array}\right], & f_{HH} & =\frac{1}{2}\left[\begin{array}{cc} 1 & -1\\ -1 & 1 \end{array}\right] \end{array}$$

(2) $$WT:\left[X_{LL},X_{LH},X_{HL},X_{HH}\right]=Conv\left(\left[f_{LL},f_{LH},f_{HL},f_{HH}\right],X\right)$$

(3) $$IWT:X=Conv^{T}\left(\left[f_{LL},f_{LH},f_{HL},f_{HH}\right],\left[X_{LL},X_{LH},X_{HL},X_{HH}\right]\right)$$

### 3.3. Overview of CWCN

**Figure 2:**
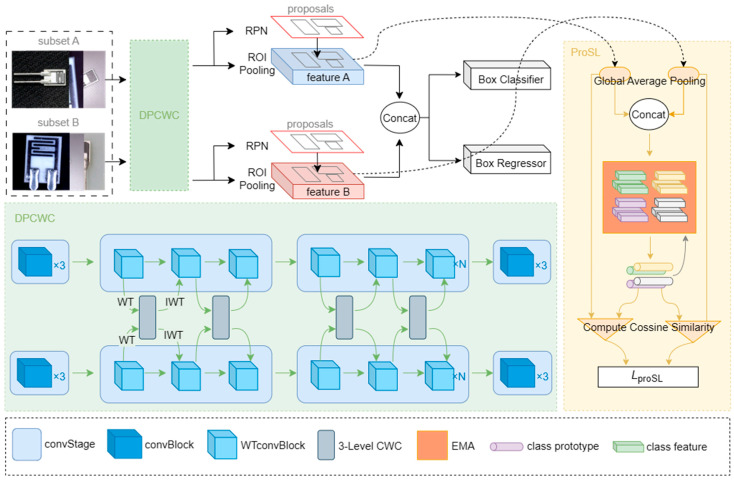
Overall framework of CWCN.

The framework of the crossed wavelet convolution network (CWCN) proposed is shown in Figure 2, which is divided into three parts, including “data preprocessing”, “feature extraction and fusion”, and “detection and loss computation”.

(1) Data preprocessing. Since our network employs a dual-pipeline feature extraction and fusion mechanism, the dataset requires special processing during model training. Specifically, the original dataset is divided into two subsets, Subset A and Subset B, both of which should contain complete and category-balanced samples. Within the same input batch, images from the two pipelines share the same category labels but exhibit different scenes. This preprocessing strategy facilitates the network in aligning features. Without such alignment, training instability may arise, potentially leading to non-convergence of the model.

(2) Feature extraction and fusion. CWCN employs a novel dual-pipeline crossed wavelet convolution training mechanism, which utilizes wavelet transform convolution (WTConv) instead of conventional convolution for feature extraction. The module also incorporates 3-level crossed wavelet convolution modules (3-Level CWC) to fuse features. The technical details are elaborated in Section 3.4.

(3) Detection and loss computation. CWCN adopts the Region Proposal Network (RPN) and ROI Pooling from Faster R-CNN [32] to process dual-pipeline feature maps. The RPN generates target proposal boxes through an anchor mechanism, while ROI Pooling maps the proposals of varying scales onto the feature maps for subsequent classification and regression. During training, the RPN loss function is computed as follows:

(4) $$L_{\mathrm{rpn}}=\frac{1}{N_{\mathrm{cls}}}\sum_{i}L(p_{i},p_{i}^{*})+\frac{\lambda }{N_{\mathrm{reg}}}\sum_{i}p_{i}^{*}\cdot L(t_{i},t_{i}^{*})$$

where $N_{\mathrm{cls}}$ and $N_{\mathrm{reg}}$ are the number of anchors, and λ is a balancing weight. $L(p_{i},p_{i}^{*})$ is the classification loss term, $p_{i}$ and $p_{i}^{*}$ represent the predicted probability and ground -truth label of the $i_{th}$ anchor, respectively. $L(t_{i},t_{i}^{*})$ is the regression loss term, where $t_{i}$ and $t_{i}^{*}$ are the predicted and ground-truth bounding box corresponding to the $i_{th}$ anchor.

Subsequently, our network concatenates the outputs from two ROI Pooling streams and employs fully connected layers to construct the predictor (consisting of a box classifier and box regressor). This process generates the classification loss $L_{\mathrm{cls}}$ and regression loss $L_{\mathrm{reg}}$ during training:

(5) $$L_{cls}=\frac{1}{N_{cls}^{’}}\sum_{j}-u_{j}log(p_{j})$$

(6) $$L_{reg}=\frac{1}{N_{reg}^{’}}\sum_{j}I(u_{j}\geq 1)\cdot \sum_{k}{smooth}_{L_{1}}(t_{j}^{k}-v_{j}^{k})$$

where $N_{cls}^{’}$ and $N_{reg}^{’}$ are the number of proposal regions, $p_{j}$ denotes the predicted class probability of $j_{th}$ ROI, and $u_{j}$ represents the ground-truth class label. The indicator function I(∗) ensures the loss is computed only for foreground samples $\left(u_{j}\geq 1\right)$. The ${smooth}_{L_{1}}(*)$ is applied to the bounding box parameters, where $t_{j}^{k}$ and $v_{j}^{k}$ correspond to the predicted and ground-truth bounding box of $k_{th}$ foreground samples.

Additionally, we design a Prototype Similarity Learning (ProSL) module to process the dual-pipeline ROI Pooling outputs, with detailed implementation described in Section 3.5.

### 3.4. DPCWC Module

The dual-pipeline crossed wavelet convolution (DPCWC) training framework proposed employs a WTConv variant as a backbone. The backbone is structured with four ConvStages, each of which contains several ConvBlocks or WTConvBlocks that perform wavelet transform (WT) and inverse wavelet transform (IWT) operations (theoretical details in Section 3.2). Compared to conventional convolution, our backbone simultaneously captures both low-frequency and high-frequency components of data features, thereby enhancing the model’s ability to perceive both global structures and local details. Unlike single-path designs, this module takes the preprocessed datasets Subset A and Subset B as dual inputs, with features being synchronously extracted through a weight-shared backbone. Consequently, our feature extraction module not only effectively decomposes global contour and local detail information of samples, but also enables the model to capture features from different scenes within the same training phase.

The dual-pipeline feature fusion follows a fundamental principle. Each pipeline preserves its global information through low-frequency components while exchanging local details via high-frequency components. Our implementation employs an alternating fusion strategy across network blocks: after performing WT in the previous block, the high-frequency components from Pipeline B are replaced by the corresponding components of Pipeline A, followed by IWT. In the subsequent block, Pipeline A’s high-frequency components are replaced by Pipeline B’s. This alternating fusion scheme continues iteratively across subsequent WTConvBlocks, where high-frequency components from Pipeline A and Pipeline B are alternately exchanged.

**Figure 3:**
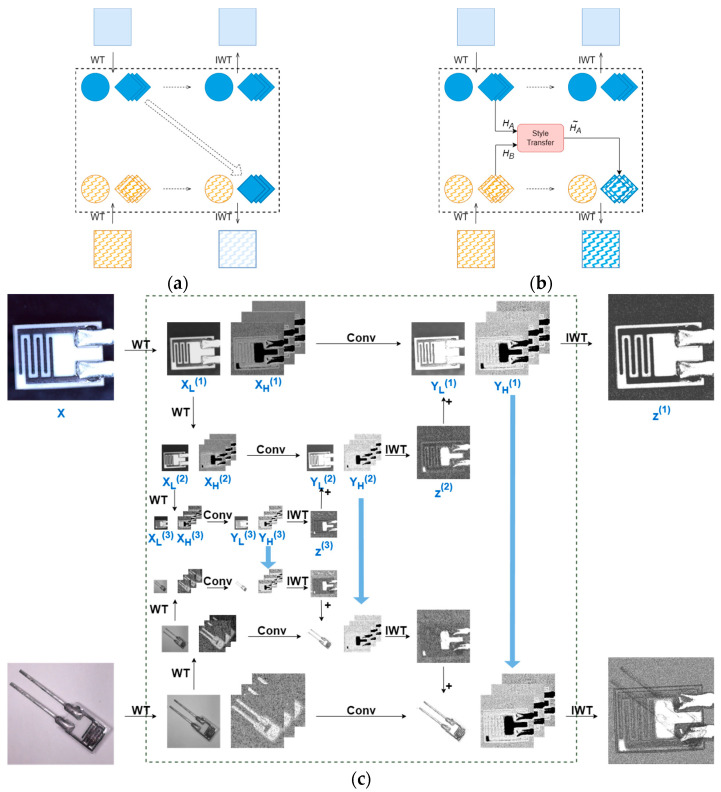
Schematic diagram of dual-pipeline feature fusion. Only the process “A to B” is shown, including (**a**) direct replacement, (**b**) style-transfer-based method, and (**c**) 3-level crossed wavelet convolution.

Based on the strategy above, we developed several implementation variants as illustrated in Figure 3.

For wavelet component processing in the dual-pipeline framework, Figure 3a demonstrates the direct replacement approach where one pipeline’s high-frequency components are simply substituted from the other. Figure 3b presents our enhanced method, building upon prior works [26,33], which performs style transfer between the high-frequency components using Equation (7) before component substitution:

(7) $$\overset{∼}{H_{A}}=\sigma (H_{B})\frac{H_{A}-\mu (H_{A})}{\sigma (H_{A})}+\mu (H_{B})$$

where μ(∗) and σ(∗) denote the mean and variance, respectively, while H and $\overset{∼}{H}$ represent the high-frequency components before and after processing, respectively.

However, we explore that the two methods above fail to adequately account for the importance of multi-scale feature fusion, resulting in significant performance disparities in detecting objects of different sizes (see Section 4.5.1 for detailed experimental results). To alleviate this impact, DPCWC adopts the 3-Level CWC method illustrated in Figure 3c. Our approach modifies the cascaded wavelet transform from [28] by implementing the following operations:

- Multi-level Wavelet Decomposition:
  ■ The input feature map X undergoes 1-level WT, yielding high-frequency component $X_{H}^{\left(1\right)}$ and low-frequency component $X_{L}^{\left(1\right)}$.
  ■ $X_{L}^{\left(1\right)}$ is further decomposed via 2-level WT to produce $X_{H}^{\left(2\right)}$ and $X_{L}^{\left(2\right)}$.
  ■ $X_{L}^{\left(2\right)}$ undergoes 3-level WT to generate $X_{H}^{\left(3\right)}$ and $X_{L}^{\left(3\right)}$.
- Convolution in Wavelet Domain:
  ■ Each level’s components perform convolutional operations in wavelet domain using Equation (8), yielding transformed components $Y_{H}^{\left(i\right)}$ and $Y_{L}^{\left(i\right)}$, $W^{\left(i\right)}$ denotes the learnable weight tensor at the level i.
- Hierarchical Reconstruction:
  ■ $Y_{H}^{\left(3\right)}$ and $Y_{L}^{\left(3\right)}$ undergo 3-level IWT to reconstruct $Z^{\left(3\right)}$.
  ■ $Z^{\left(i\right)}$ is combined with $Y^{\left(i-1\right)}$ through element-wise addition to form the composite low-frequency component at the level (i−1)
  ■ Feature maps at each level are reconstructed by applying IWT to the composite low-frequency components and corresponding $Y_{H}^{\left(i-1\right)}$, as formalized in Equation (9).
- Cross-pipeline Feature Fusion:
  ■ During synchronous dual-pipeline processing, high-frequency components ($Y_{H}^{\left(1\right)}$, $Y_{H}^{\left(2\right)}$, and $Y_{H}^{\left(3\right)}$) from one pipeline replace their corresponding counterparts in the other.
  ■ The modified components then undergo cascaded IWT operations.
    (8) $$Y_{L}^{\left(i\right)},Y_{H}^{\left(i\right)}=Conv(W^{\left(i\right)},(X_{L}^{\left(i\right)},X_{H}^{\left(i\right)}))$$
    (9) $$Z^{\left(i-1\right)}=IWT(Y_{L}^{\left(i-1\right)}+Z^{\left(i\right)},Y_{H}^{\left(i-1\right)})$$

We also develop a stage-wise fusion strategy, enabling the 3-Level CWC to carry out more effective multi-scale feature fusion and information interaction. Specifically, when applied to shallower network layers, 3-Level CWC excels at integrating fine-grained features, while its application in deeper layers facilitates the fusion of coarse-grained features. However, DPCWC does not employ this fusion operation across all four ConvStages of the backbone but rather selectively implements 3-Level CWC in the second and third ConvStages. This strategic design is based on three key considerations: First, it simultaneously addresses fusion requirements for both shallow and deep network features, ensuring comprehensive multi-scale representation throughout the network hierarchy. Second, it prevents excessive cross-pipeline interference and maintains distinctive feature characteristics in each pipeline, reducing the risk of feature homogenization. Third, it avoids unnecessary parameter proliferation to control model complexity. As evidenced by the experimental results in Section 4.5.2, our selective fusion approach achieves an optimal balance between representation learning capability and computational efficiency.

### 3.5. ProSL Module

For the cross-scene features from the two pipelines, a dedicated loss computation module is needed to establish and reinforce feature correlations, thereby guiding the model to learn more generalizable feature representations. If our loss computation module appropriately processes the dual ROI Pooling outputs, which contain both categorical and spatial coordinate information from multi-scene, the model can be optimized for category classification and object localization simultaneously.

**Figure 4:**
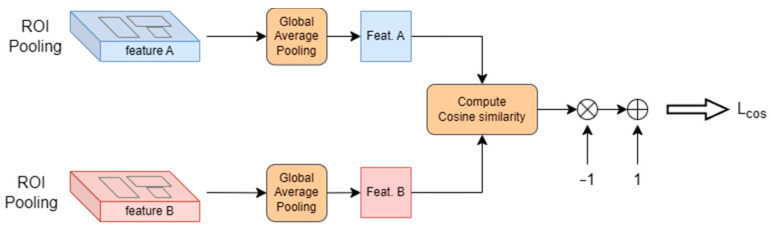
Self-supervised loss computation module.

Inspired by the contrastive learning framework of SimCLR [34], we initially designed a self-supervised loss computation module. As illustrated in Figure 4, the module first applies global average pooling (GAP) to reduce dimension and computational complexity. The low-dimensional feature vectors are then used to compute cosine similarity by Equation (10):

(10) $$Sim.(FeatA,FeatB)=\frac{\sum_{i=1}^{n}FA_{i}*FB_{i}}{\sqrt{\sum_{i=1}^{n}FA_{i}^{2}}\sqrt{\sum_{i=1}^{n}FB_{i}^{2}}}$$

where the numerator represents the dot product of the two feature vectors and the denominator is the product of their Euclidean norms. To promote similar feature distributions between the two pipelines, the model optimization aims to minimizes the loss value in Equation (11).

(11) $$L_{cos}=1-Sim.(FeatA,FeatB)$$

However, this loss computation is inherently limited to contrastive learning between the dual-pipeline features. While the trained model achieves cross-scene feature alignment and effectively determines whether samples from Subset A and B belong to the same category, the inter-class feature clustering relies solely on the Faster R-CNN’s loss computations (Equations (4)–(6)), which leads to insufficient discriminative capability between different categories. In our chip defect detection tasks, this limitation manifests defect misclassification with similar visual characteristics, ultimately failing to address the practical requirements of defect detection.

Building upon the aforementioned loss computation module, we propose Prototype Similarity Learning (ProSL) as illustrated in Figure 2. ProSL incorporates category label information as prior knowledge to guide model learning, so it falls under the supervised learning (SL) paradigm. The computational details of this module are presented in Algorithm 1.

In ProSL module, the dual-pipeline features from ROI Pooling undergo dimensionality reduction and concatenation, resulting in ${Feat}_{A\&B},$ which contains features of all ROIs from both Subset A and B. ${Feat}_{A\&B}$ is then utilized to construct prototypes through the following procedure: features are grouped according to category labels and Intersection over Union (IoU), yielding class-specific features, and then the mean features are computed as prototypes. All the category prototypes including background type are refined using Exponential Moving Average (EMA) as shown in Equation (12):

(12) $${Proto}_{i}=(1-\beta )\times {Proto}_{i-1}+\beta \times {Proto}_{init}$$

where ${Proto}_{i-1}$ and ${Proto}_{i}$ denote prototypes of the previous and current batch. In the first batch, ${Proto}_{i-1}$ (i.e., ${Proto}_{0}$) is constructed by a pre-trained model and all the support samples. ${Proto}_{init}$ is an initial model created by the current ${Feat}_{A\&B}$. β is the momentum coefficient, whose initial value is 0.1 during the “warm-up” phase and decreases to 0.01. This momentum-based update strategy establishes robust prototypes during initial training epochs while subsequently smoothing the prototype evolution, mitigating excessive fluctuations caused by distribution shifts within individual training batch.
**Algorithm 1.** ProSL$Require:\;\mathrm{ROI}\;\mathrm{pooling}\;\mathrm{outputs}\;\mathrm{after}\;\mathrm{dimensional}\;\mathrm{reduction}\;{Feat}_{A}$$\;\mathrm{and}\;{Feat}_{B}$$,\;\mathrm{pre}\text{-}\mathrm{prototypes}\;{Proto}_{0}$ created by pretrained model and all support samples$1:\;Feat_{A\&B}\leftarrow \mathrm{Cat}({Feat}_{A},{Feat}_{B})$ //Concatenate feature maps to dimension (N_A_+N_B_, D)$2:\;\left(k+1\right)\;groups\;off\;eats\;\leftarrow \;\mathrm{Group}(Feat_{A\&B})$ //Group features by annotations for each category, containing background type$3:\;for\;i\;\in \;\left\{0,1,\ldots K\right\}\;do$$4:\;Create\;every\;category\;prototype\;P_{i}\;using\;Equation\;\left(12\right)\;based\;on\;{Proto}_{0}$5: end for$6:\;Proto\leftarrow \mathrm{Cat}(P_{i})$ // Compute the final prototypes for current batch to dimension (K, D)$7:\;for\;j\;\in \;\left\{A,B\right\}do$$8:\;Compute\;{Sim}_{j}\;between\;{Feat}_{j}\;and\;Proto\;using\;Equation\;(10)$ //Dimension is (N_j_, K)9: end for$10:\;L_{\mathrm{ProSL}}\leftarrow \mathrm{ComputeLoss}\left(Sim_{A},Sim_{B}\right)via\;Equation\;(13)$$11:\;return\;L_{\mathrm{ProSL}}$

Subsequently, the dual-pipeline features ${Feat}_{A}$ and ${Feat}_{B}$ are computed similarity with the prototypes ${Proto}_{i}$ using Equation (10) to obtain ${Sim}_{A}$ and ${Sim}_{B}$, respectively. The final loss objective of ProSL is to minimize the value in Equation (13), where τ is 0.1 as a scaling factor, ${sim}^{pos}$ is the similarity of positive sample and ${sim}^{k}$ denotes the similarity for each category.

(13) $$L_{ProSL}=-\log\left(\frac{\exp\left(\frac{{sim}_{A}^{pos}}{\tau }\right)}{\sum_{k=1}^{K}\exp\left(\frac{{sim}_{A}^{k}}{\tau }\right)}\right)-log\left(\frac{\exp\left(\frac{{sim}_{B}^{pos}}{\tau }\right)}{\sum_{k=1}^{K}\exp\left(\frac{{sim}_{B}^{k}}{\tau }\right)}\right)$$

(14) $$L_{total}=L_{rpn}+L_{cls}+L_{reg}+L_{ProSL}$$

The loss value $L_{ProSL}$ is combined with the base losses from Equations (4)–(6) to form the total optimization objective $L_{total}$, which is backpropagated to jointly optimize the entire model.

We conducted ablation studies evaluating the effectiveness of different loss functions to identify the optimal module for CWCN (results shown in Section 4.5.3). Based on the special design of DPCWC, we ultimately adopt the ProSL loss module to strengthen the model’s ability to learn inter-pipeline feature correlations while preserving category discriminability. This option enables the model to better adapt to cross-domain few-shot chip defect detection tasks under multi-scene conditions.

## 4. Results and Discussion

### 4.1. Datasets

In our work, we use COCO2014 [35] as the source domain dataset, and the dataset processing approach is based on [19] as a reference, where 98,459 images covering 60 classes are used for model pretraining.

Our method focuses on industrial chips of a humidity sensor. Two customized chip datasets are collected for performance comparison: ChipNormal (single-scene) and ChipMix (multi-scene). ChipNormal contains images captured solely under normal lighting conditions, while ChipMix incorporates multiple scenarios, as shown in Figure 1b. High-resolution images are captured by a CCD camera with a resolution of 5120 × 2880, while the other two scenes have a resolution of 1920 × 1080. Night-vision images are captured by an infrared camera with a wavelength of 840 nm. The two datasets have the same defect categories, as shown in Figure 1a, both being divided by the same strategy, including 3-way-1-shot, 3-way-5-shot, and 3-way-10-shot support sets for fine-tuning. We use data augmentation (e.g., rotation, cropping, etc.) to create the query set, obtaining 150 images for each scene. The types of defects in each scene are relatively balanced. Incidentally, ChipNormal uses only 150 images captured under normal lighting conditions, whereas ChipMix consists of a total of 450 images.

To further validate our method, we also use an open-source dataset NEU-DET [36] as the target domain dataset for comparison experiments. This dataset focuses on steel surface defects, including six common defect types, and we refer to [22] to create 6-way-1-shot, 6-way-5-shot and 6-way-10-shot support sets, along with 360 images as a query set.

### 4.2. Evaluation Metric

In object detection, Average Precision (AP) serves as a key evaluation index for assessing model performance. AP calculation involves fundamental concepts:

(15) $$Precision=\frac{TP}{TP+FP}$$

(16) $$Recall=\frac{TP}{TP+FN}$$

(17) $$\mathrm{IoU}=\frac{\mathrm{Intersection}\;\mathrm{Area}}{\mathrm{Union}\;\mathrm{Area}}=\frac{A\cap B}{A\cup B}$$

True Positives (TPs) represent correctly detected positive samples, False Positives (FPs) denote incorrectly identified positive samples, and False Negatives (FNs) indicate missed positive samples. IoU is short for “Intersection over Union”, and A and B correspond to the areas of predicted bounding boxes and ground truth boxes, respectively.

AP is computed as the area under the Precision–Recall curve (PR curve), which comprehensively reflects the model’s precision performance across varying recall rates. Our work employs mean Average Precision (mAP) as the primary evaluation metric, computed according to the COCO standard [35]. For individual categories, the AP value is calculated across multiple IoU thresholds (ranging from 0.5 to 0.95 with a step size of 0.05, totaling 10 thresholds) and then averaged to obtain ${\mathrm{AP}}_{c}$. The mAP is subsequently derived as the mean of ${\mathrm{AP}}_{c}$ across all categories (as formulated in Equation (18)).

(18) $$\mathrm{mAP}=\frac{1}{N_{c}}\sum_{c=1}^{N_{c}}{\mathrm{AP}}_{c}=\frac{1}{N_{c}}\sum_{c=1}^{N_{c}}\;\frac{\sum_{IoU}{\mathrm{AP}}_{\mathrm{IoU}}}{N_{\mathrm{IoU}}}$$

Additionally, following the PASCAL VOC standard [37], we adopt single-IoU-threshold precision metrics AP50 and AP75, where detections are considered correct if their IoU with ground truth exceeds 50% or 75%, respectively. To assess the performance on multi-scale objects, the evaluation also includes APs (Small Object AP), APm (Medium Object AP), and APl (Large Object AP).

To specifically evaluate the model’s cross-scene performance in chip defect detection, we introduce a novel index, $Gap_{AP}$, defined as the mAP gap between models trained on different datasets (ChipNormal versus ChipMix), i.e., a smaller $Gap_{AP}$ indicates superior model adaptability across diverse scenarios.

### 4.3. Experimental Setup

To make a fair comparison, the pretraining settings of all implementations followed [19] and our method employ one pipeline without ProSL for pretraining.

In the fine-tuning phase, all implementations adopt full-parameter fine-tuning. The learning rate schedule follows a warm-up and decay strategy: 10% of the total epochs are “warm-up” trained at a learning rate of 1 × 10^−5^, followed by 55% epochs at a base learning rate (BASE_LR). The learning rate is then decayed to 10% BASE_LR for 30% epoch training, and finally reduced to 1% for the remaining epochs. For all k-shot tasks on the chip datasets, we set around 80 training epochs, with a BASE_LR of 0.001; for the open-source dataset, the settings followed the work of [22], tuning on 1-shot tasks around 80 epochs and on 5/10-shot tasks around 40 epochs, with the BASE_LR of 0.002. All experiments are conducted on a GeForce RTX 3090.

It should be noted that in certain cases, such as 1-shot tasks, dividing the data into two subsets may not be feasible. To ensure both pipelines process the same categories for effective feature alignment, we employ only one pipeline for the first epoch, and thereafter utilize components of the previous epoch into pipeline B of the current epoch.

### 4.4. Results and Analyses

#### 4.4.1. Results on Chip Datasets

Table 1, Table 2 and Table 3 present the results of different algorithms on both ChipNormal and ChipMix datasets. The vertical comparison among algorithms demonstrates that our method consistently outperforms others across various k-shot tasks, achieving superior performance in both single-IoU-threshold metrics (AP50, AP75) and the more rigorous mAP criterion. Horizontal analysis across datasets reveals that our approach exhibits smaller $Gap_{AP}$ values compared to other methods. This strongly validates the enhanced scenario adaptability of our proposed method.

**Figure 5:**
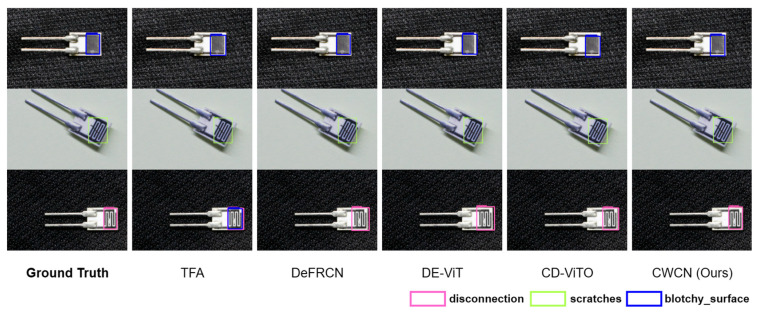
Visual comparison of prediction results. Three-way-ten-shot detection on ChipNormal is shown.

**Figure 6:**
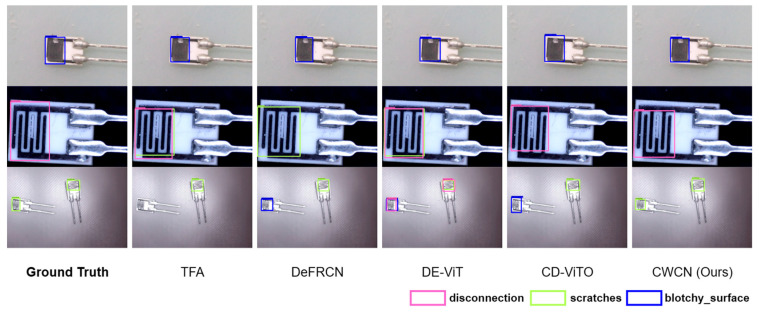
Visual comparison of prediction results. Three-way-ten-shot detection on ChipMix is shown.

Figure 5 and Figure 6 visualize the prediction results (confidence threshold is 0.5). The comparative visualizations reveal that all comparison methods perform consistently on single-scene datasets. As for ChipMix, while other methods exhibit either false positives or missed detections, our method demonstrates significantly enhanced robustness when handling defect samples across different scenarios.

Analyzing theoretically, existing methods face two fundamental challenges when processing mixed-scene data: one is ineffective fusion of cross-scene semantic information, and the other is inter-scenario interference that makes it harder to learn feature representations from any individual scene. In contrast, our method addresses these limitations through dual-pipeline feature extraction and wavelet-based component fusion, further enhanced by the ProSL module to strengthen feature consistency.

#### 4.4.2. Results on NEU-DET

To comprehensively evaluate models’ performance, we conducted comparative experiments on the open-source NEU-DET dataset from two perspectives: (1) comparison among different methods and (2) comparison across backbones of varying scales.

**Figure 7:**
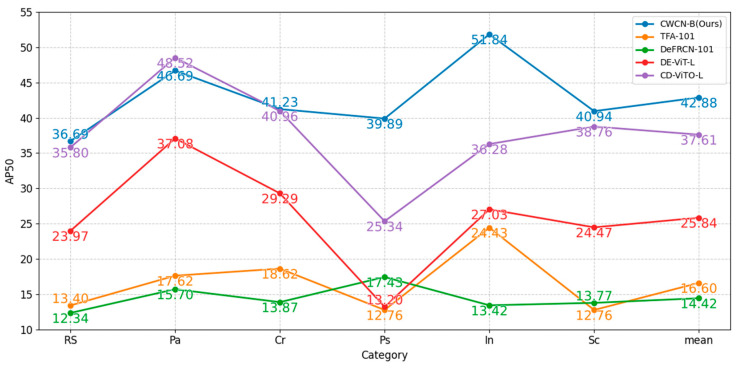
AP50 results for all the categories on NEU-DET. Six-way-ten-shot detection is shown. The best indicators are shown in bold.

The results in Table 4 demonstrate that our method achieves superior mAP across all few-shot configurations, with performance improvement observed as the backbone scale increases. This means that larger backbones in CWCN possess enhanced feature extraction capabilities. Table 4 also reports the frames per second (FPS) for 10-shot tasks, demonstrating that our method achieves competitive inference speed, with the lightweight variant exhibiting superior efficiency. Furthermore, Figure 7 shows the AP50 of the comparison methods in each category, and the results demonstrate that our method performs relatively steadily in inter-class detection.

Unlike conventional CNNs or Transformers, our WTConv-enhanced backbone conducts convolutions in the wavelet domain rather than direct spatial convolution or attention mechanism, gradually expanding receptive fields without over-parameterization via multi-level cascaded wavelet blocks [28]. As evidenced by the experiments, the whole CWCN architecture, integrating this advanced backbone with dual-pipeline design and specialized loss computation, demonstrates generalizability beyond industrial chips. This method exhibits potential to defect inspection tasks across various industrial workpieces.

### 4.5. Ablation Study

#### 4.5.1. Analysis of Feature Fusion Modules

To validate the 3-Level CWC, we conducted an ablation study in Table 5, which reveals significant limitations in two initial approaches. Direct Replacement achieves only 29.75% mAP overall, particularly deficient in small object detection (APs = 19.82%). The Style-Transfer-Based Method shows moderate improvement but still exhibits substantial scale imbalance with the gap between 51.98% APl and 22.26% APs.

In contrast, our 3-Level CWC demonstrates consistent performance gains across all evaluation metrics. This advancement primarily stems from its multi-scale fusion mechanism, which better accommodates varying object sizes through hierarchical wavelet decomposition and adaptive cross-pipeline component interaction.

#### 4.5.2. Analysis of Selective Staged Fusion Strategy

Regarding the question of which ConvStage the 3-Level CWC should be applied in DPCWC, we conducted relevant experiments. Empirically, the crossed wavelet transform needs to be applied in both shallower and deeper layers to fuse multi-scale information. Thus, we conduct several schemes as shown in Table 6. The results show that excessive fusion (Scheme 2) leads to performance degradation, as frequent cross-pipeline interactions introduce feature interference that compromises each pipeline’s distinctive feature representations. Additionally, although Scheme 3 achieves precision comparable to our method, it does so at more complicated training parameters.

**Figure 8:**
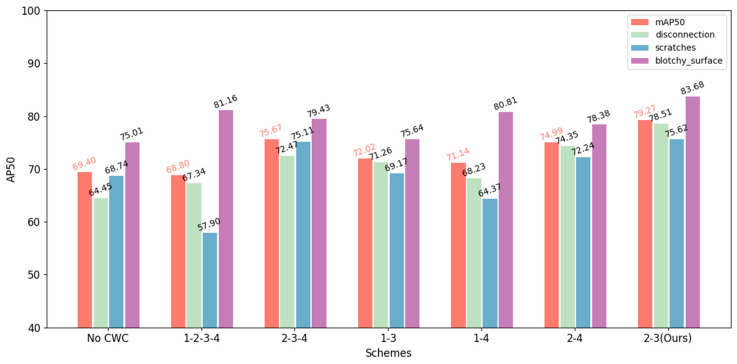
Category AP50 of different selective schemes. Based on 3-way-10-shot on ChipMix.

To further evaluate the rationality of our selective scheme, the results of AP50 at the category level are shown in Figure 8. Among them, over-fusion schemes may confuse more visual characteristics in shallower ConvStages. It increases the difficulty of training, manifesting performance degradation and fluctuation across categories. Properly integrating encoded features rather than visual features avoids these situations.

The proposed 2nd-3rd-ConvStage scheme emerges as the optimal solution, effectively balancing multi-scale feature fusion with computational efficiency. This selective fusion strategy successfully captures both local textures and global structures while preserving pipeline-specific characteristics without significantly increasing computational overhead.

#### 4.5.3. Analysis of Loss Calculation Modules

**Figure 9:**
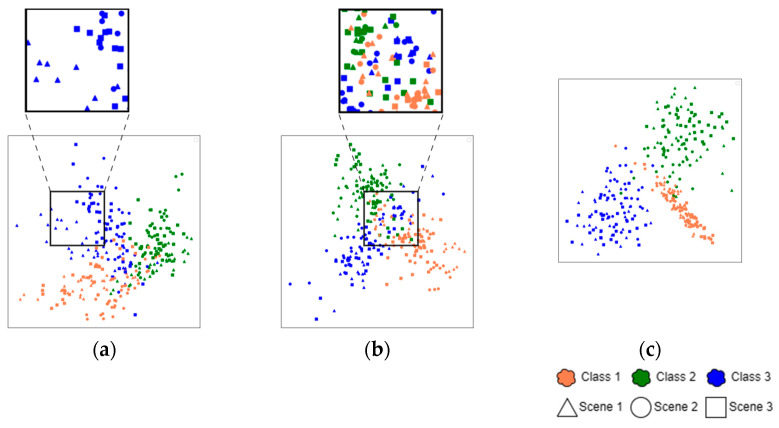
T-SNE visualization. (**a**) Baseline, (**b**) SSL module, and (**c**) ProSL (ours).

To evaluate different loss computation modules, we conducted a comparative visualization experiment. The same query samples were processed through feature extractors equipped with different loss modules to obtain high-dimensional representations, which were then projected into a 2D space using t-SNE dimensionality reduction. The visualization results in Figure 9 depict feature points of three scenarios (represented by triangles, circles, and squares, respectively) and three defect categories (colored coral, green, and blue). As our study focuses on two key aspects:

- The overlap between different geometric markers indicates cross-scene adaptability. More overlap suggests better scenario-invariant feature learning.
- The separation between color-coded clusters reflects category discriminability. Clearer boundaries mean stronger classification capability.

When solely on Faster R-CNN’s original loss functions, it is difficult to adapt to CWCN’s dual-pipeline design, as shown in Figure 9a, due to insufficient inter-class discrimination and poor cross-scene feature alignment. The visualization result proves the necessity of a specialized loss computation module. Figure 9b displays the feature distribution obtained using the self-supervised loss module, with substantial overlapping areas between different-colored points. While this approach achieves partial success in clustering feature points from different scenarios, it still suffers from inter-class confusion. This limitation stems from the module’s inherent design flaw, it focuses exclusively on inter-pipeline feature comparison while lacking explicit category discrimination guidance. It remains dependent on Faster R-CNN’s original loss functions for classification and localization.

In contrast, the model employing the ProSL module (Figure 9c) demonstrates superior distribution characteristics. The features show tighter intra-class clustering and clearer inter-class separation boundaries while effectively aligning same-class samples across different scenarios. This distribution pattern visually confirms ProSL’s dual advantage in maintaining both scenario consistency and category specificity.

### 4.6. Discussion

Our method has successfully achieved cross-domain, multi-scene industrial chip defect detection under few-shot conditions.

However, the results of APs, APm, and Apl reveal notable performance fluctuations when detecting defects of different sizes. Taking the 10-shot task on ChipMix as an example, Table 3 reports that our method achieves 67.28% in Apl, while showing lower performance in APm (41.64%). Although these indicators show improvement over the baselines, the size-dependent performance gap appears more pronounced in our method than in others.

The potential reasons for this limitation are as follows: our backbone only ensures multi-scale feature fusion between the two pipelines. But it neglects the importance of the pipeline itself in multi-scale feature extraction. CWCN’s single pipeline does not use technologies such as FPN, which is an idea worth exploring in the future.

Additionally, our method is currently limited to the two-stage object detection framework (Faster R-CNN) and it satisfies offline batch processing but falls short of real-time demands in online detection. Thus, adapting our approach to one-stage frameworks is a key focus of our ongoing research.

## 5. Conclusions

Our work focuses on defect detection in industrial chips, thoroughly investigating the challenges of cross-domain few-shot learning under multi-scene conditions. We propose the crossed wavelet convolution network (CWCN), whose core components include (1) a novel dual-pipeline crossed wavelet convolution (DPCWC) training mechanism that achieves multi-scene feature alignment through wavelet component extraction and a selective fusion strategy; (2) a Prototype Similarity Learning (ProSL) module that employs class prototypes and pipeline features similarity calculation, enforcing scenario consistency while enhancing feature discriminability. Comprehensive comparative experiments and ablation studies confirm the effectiveness of our method in improving both cross-scene adaptability and detection accuracy. However, limitations remain: the approach shows room for improvement in handling multi-scale defects, and its performance in online industrial detection requires further investigation.

Our work expands the application boundaries of wavelet transform. While wavelet transform has been extensively studied and applied in signal processing, its exploration in computer vision and image processing remains relatively limited. Our work not only provides a novel case for applying wavelet transform in image processing but also represents a successful example of introducing wavelet transform convolution into few-shot learning, particularly for cross-domain few-shot object detection.

## Figures and Tables

**Table 1:** Results of 1-shot detection task on chip datasets. The best indicators are shown in bold, and the down arrow means that lower scores have better quality.

Method	Dataset												Gap_AP_ ↓
	ChipNormal						ChipMix						
	mAP	AP50	AP75	APs	APm	APl	mAP	AP50	AP75	APs	APm	APl	
TFA [19]	36.02	64.51	33.13	19.53	51.17	37.35	18.75	37.65	14.47	14.63	21.22	20.40	17.27
DeFRCN [20]	38.23	62.85	41.14	31.25	40.63	42.80	19.20	33.01	21.19	15.05	18.52	24.03	19.03
DE-ViT [21]	44.17	63.83	47.36	37.60	**54.59**	40.33	25.73	37.14	25.03	13.60	25.39	38.19	18.44
CD-ViTO [22]	**50.04**	68.38	60.61	**45.49**	49.20	55.42	23.53	37.75	29.19	18.51	28.02	24.07	26.51
CWCN (Ours)	47.84	**73.53**	**66.01**	33.19	50.42	**59.91**	**39.96**	**52.44**	**39.01**	**34.02**	**33.13**	**52.74**	**7.88**

**Table 2:** Results of 5-shot detection task on chip datasets. The best indicators are shown in bold, and the down arrow means that lower scores have better quality.

Method	Dataset												Gap_AP_ ↓
	ChipNormal						ChipMix						
	mAP	AP50	AP75	APs	APm	APl	mAP	AP50	AP75	APs	APm	APl	
TFA [19]	50.57	80.25	58.14	37.59	57.95	56.18	30.51	59.26	28.99	27.86	30.67	32.99	20.06
DeFRCN [20]	50.07	78.25	56.21	36.50	56.57	57.13	33.58	47.31	40.52	30.90	39.49	30.33	16.49
DE-ViT [21]	48.01	68.75	54.70	**49.92**	51.65	42.46	36.88	52.13	44.80	26.80	33.98	49.88	11.13
CD-ViTO [22]	49.64	70.28	62.76	41.17	56.48	51.28	37.41	52.01	46.61	30.83	35.50	45.90	12.23
CWCN (Ours)	**54.48**	**83.55**	**65.61**	24.06	**65.57**	**73.81**	**47.59**	**78.29**	**55.92**	**36.88**	**51.61**	**54.29**	**6.89**

**Table 3:** Results of 10-shot detection task on chip datasets. The best indicators are shown in bold, and the down arrow means that lower scores have better quality.

Method	Dataset												Gap_AP_ ↓
	ChipNormal						ChipMix						
	mAP	AP50	AP75	APs	APm	APl	mAP	AP50	AP75	APs	APm	APl	
TFA [19]	60.85	90.20	70.07	**55.3** **0**	64.97	62.28	38.45	68.30	40.05	35.82	37.69	41.85	22.40
DeFRCN [20]	58.48	**94** **.00**	59.35	49.82	59.00	66.62	37.35	71.71	33.09	29.65	40.82	41.56	21.13
DE-ViT [21]	57.05	76.88	63.17	52.33	53.99	64.83	39.61	54.93	47.22	25.75	44.32	48.76	17.44
CD-ViTO [22]	61.10	80.98	76.84	43.67	**77.14**	62.48	39.09	56.58	47.69	30.50	**46.47**	40.29	22.01
CWCN (Ours)	**63.86**	88.29	**82.53**	49.09	70.59	**71.91**	**50.22**	**79.27**	**55.69**	**41.73**	41.64	**67.28**	**13.64**

**Table 4:** Results of k-shot detection task on NEU-DET. The best indicators are shown in bold.

Model	Backbone	1-Shot	5-Shot	10-Shot	
		mAP	mAP	mAP	FPS
TFA [19]	ResNet50	0.00	4.50	4.85	19.72
	ResNet101	0.25	4.64	5.65	15.43
DeFRCN [20]	ResNet50	0.00	4.20	5.29	9.59
	ResNet101	0.81	4.76	5.67	8.66
DE-ViT [21]	Vit-B	0.38	5.99	6.74	7.97
	Vit-L	0.68	7.61	8.86	4.15
CD-ViTO [22]	Vit-B	3.92	10.84	13.03	7.73
	Vit-L	3.99	10.68	12.45	3.98
CWCN (Ours)	WTConvNeXt-T	4.32	10.31	11.18	**19.93**
	WTConvNeXt-S	4.87	11.98	13.22	15.51
	WTConvNeXt-B	**5.13**	**12.27**	**14.68**	9.10

**Table 5:** Verification of feature fusion module. Based on 3-way-10-shot on ChipMix. The best indicators are shown in bold.

Method	mAP	APs	APm	APl
Direct Replacement	29.75	19.82	38.48	41.26
Style-Transfer-Based Method	43.45	22.26	39.51	51.98
3-Level CWC	**50.22**	**41.73**	**41.64**	**67.28**

**Table 6:** Verification of selective staged fusion strategy. Based on 3-way-10-shot on ChipMix. The best indicators are shown in bold, and the down arrow means that lower scores have better quality.

Serial No.	Method	mAP	Parameters ↓
1	No CWC	45.63	baseline
2	1st-2nd-3rd-4th	44.94	+5M
3	2nd-3rd-4th	49.84	+4M
4	1st-3rd	47.51	+2M
5	1st-4th	46.34	**+1M**
6	2nd-4th	46.15	**+1M**
7	2nd-3rd (Ours)	**50.22**	+2M

## Data Availability

Currently, the datasets in this work are part of ongoing research and they will be available upon request from the corresponding author.
